# A Wearable TENS Garment for Joint Pain Management: IEC 60601 Compliant Design and Preliminary Evaluation

**DOI:** 10.1109/JTEHM.2026.3663967

**Published:** 2026-02-11

**Authors:** Irfan Ullah, Tyler Ward, Tom Greig, Gillian Lake-Thompson, Meijing Liu, Lynn Reeves, Elaine Dennison, John Tudor, Kai Yang

**Affiliations:** School of Electronics and Computer ScienceUniversity of Southampton 7423 SO17 1BJ Southampton U.K.; Winchester School of ArtUniversity of Southampton 7423 SO23 8DL Winchester U.K.; MRC Lifecourse Epidemiology CentreUniversity of Southampton 7423 SO16 6YD Southampton U.K.

**Keywords:** Transcutaneous electrical nerve stimulation (TENS), osteoarthritis, joint pain, electrode, E-textiles, wearable medical devices, IEC 60601 compliance, MHRA, home usability, electrotherapy, pain management, clinical trial

## Abstract

Objectives: To develop and evaluate a wearable, garment-integrated transcutaneous electrical nerve stimulation (TENS) system for relieving osteoarthritis knee pain, emphasizing safety, usability, and readiness for home and clinical deployment.Methods: We designed an IEC 60601 compliant TENS system that embeds flexible electrodes into a close-fitting, machine-washable textile. A seven-day, home-based usability evaluation was conducted with 11 participants with osteoarthritis. Outcomes included self-reported pain (baseline vs. post-use) and usability metrics (ease of setup and comfort). The system received Medicines and Healthcare products Regulatory Agency (MHRA) and Health Research Authority (HRA) approvals for a subsequent clinical investigation.Results: Participants reported strong user acceptance, ease of use and comfort. Average pain decreased by 54.79% over the evaluation period, indicating a meaningful short-term analgesic benefit in a home setting. No serious adverse events were observed.Conclusion: Integrating electrodes into a wearable garment addresses key limitations of conventional adhesive-pad TENS, improving placement consistency, comfort, and ease of use while supporting safe operation under IEC 60601. These preliminary findings support the feasibility of garment-based TENS for osteoarthritis management at home and justify a follow-on clinical trial to rigorously quantify pain relief, functional outcomes, and user satisfaction in a larger cohort. Clinical Impact: The use of a washable TENS garment, compliant with IEC 60601, resulted in reduced osteoarthritis pain in a home setting. Its integration into home care is facilitated by an easy to use device with reusable textile electrodes

## I. Introduction

**FIGURE 1.**
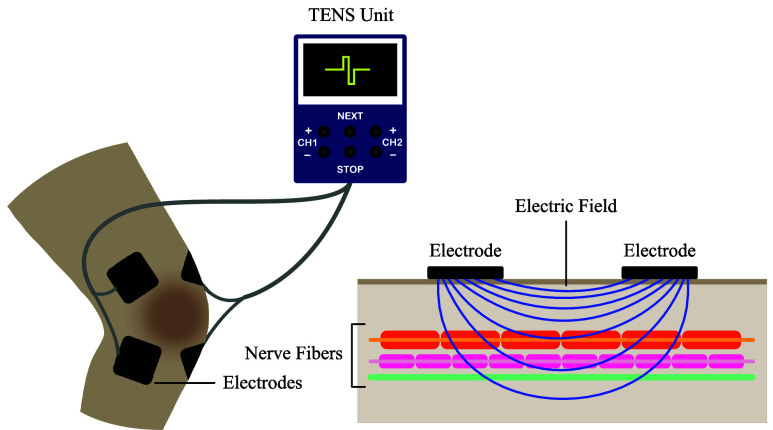
Conceptual diagram of a TENS system. Electrodes placed on the skin deliver a controlled electrical current from the TENS unit to provide impulses to the underlying nerves, helping reduce pain by promoting endorphin release and interrupting pain signals.

Osteoarthritis is a leading cause of disability worldwide, resulting in chronic joint pain and significant functional limitations, including reduced mobility and impaired performance of daily activities [1]. Approximately 595 million people worldwide were affected by osteoarthritis in 2020, representing 7.6% of the world’s population. This is a significant increase of 132% in total cases since 1990 [2]. An aging global population will exacerbate this. Furthermore, compared to the cases reported in 2020, the prevalence of osteoarthritis is projected to rise by 74.9% for knee osteoarthritis by 2050 [2]. Both pharmacological and non-pharmacological interventions are commonly used to alleviate symptoms and improve mobility in osteoarthritis patients. Among non-pharmacological therapies, Transcutaneous Electrical Nerve Stimulation (TENS), illustrated in Fig. 1, has gained considerable attention for its ability to stimulate the sensory nerves around affected joints and reduce pain perception [3], [4], [5].

Conventional TENS devices typically use adhesive gel electrodes that are individually manually placed on the skin [6], [7], [8]. This approach presents challenges for patients with limited dexterity or mobility, such as older adults with arthritis; poorly placed or dislodged electrodes can compromise therapeutic effectiveness and hence reduce patient adherence and outcomes. Additionally, disposable adhesive electrodes, although widely used, incur substantial recurring costs that create financial barriers to long-term therapy. Moreover, they also generate considerable medical waste and contribute to environmental pollution.

Recent advancements in wearable medical technologies have addressed these issues by integrating reusable electrodes into textiles, ensuring a consistent contact location, reducing waste, and simplifying the application process [9], [10], [11]. When tailored to the needs of osteoarthritis patients, wearable TENS garments can provide optimized and secure electrode placement around the affected joints, making them a convenient and reliable way to provide stimulation. These garments can also undergo multiple machine laundering cycles without losing functionality, facilitating long-term reuse and significantly reducing recurring costs and medical waste compared to disposable gel electrodes. Additionally, garment-based electrodes aim to maintain clinical reliability during movement, marking another improvement over gel electrodes which are prone to detaching, particularly after several uses.

This transition to integrated wearable TENS addresses three critical healthcare priorities: (i) cost-effective chronic pain management, (ii) environmentally responsible medical technology, and (iii) accessible design for aging populations. By overcoming the key limitations of conventional electrodes, wearable integration transforms TENS from an onerous treatment into a practical, sustainable solution for managing osteoarthritis pain.

While numerous TENS prototypes have been reported, they are predominantly proof-of-concept platforms which are not optimized for integrated, safe clinical use. Prior examples include IoT-based assemblies built from development boards [12], [13], which lack a medical grade enclosure and garment integration; a compact closed-loop platform [14] that omits formal safety compliance; and a portable device and a flexible laboratory stimulator [15], [16] that are not designed for wearable applications. Moving beyond these limitations, our work introduces a compact, single-PCB TENS unit specifically designed to dock into a knee garment, distinguished by its safety-by-design approach grounded in IEC 60601 and ISO 14971, and by its iterative co-design and testing in a lab setting followed by evaluation through an early-stage home usability study with knee osteoarthritis patients. This combination of ergonomic garment integration, formal safety considerations and usability testing advances the prior state of the art towards a deployable medical device for knee OA.

Building on this, we present a novel wearable TENS garment designed to alleviate joint pain in osteoarthritis patients by integrating flexible electrodes directly into the textile. This configuration enables targeted stimulation of pain area(s) without the need for placing each electrode individually. We describe the electronics hardware design ensuring compliance with the IEC 60601 standard, and discuss the process for securing MHRA approval, emphasizing key design choices to ensure user’s needs and regulatory requirements as defined in the standards, are met. A custom-designed TENS device was required to provide the necessary high-output, dual-mode stimulation, enhanced 2 Means Of Patient Protection (MOPP) safety for at-home use, simplified user controls, and a credible sham mode for future randomized controlled trials. The key standards relevant to this device are:

1) IEC 60601-1 which details the safety and performance requirements common to all types of medical devices [17].
2) IEC 60601-1-2 which contains the requirements related to electromagnetic compatibility [18].
3) IEC 60601-1-6 which lists the requirements for device usability [19].
4) IEC 60601-1-11 which gives the additional requirements placed on devices which are used in the ‘home healthcare environment’ defined as any environment where healthcare professionals are not continually available [20].
5) IEC 60601-2-10 which supplements and occasionally overrides previous standards with requirements specific to electrical, nerve and muscle stimulators [21].

It is common for one standard to refer to another as a way of including requirements that are more general in scope than the standard itself. IEC 60601, IEC 62304 [22] and IEC 82304 [23] are used to define the requirements for the software development process; IEC 60086 [24] and IEC 60529 [25] are used to give details of the environmental testing procedures. In addition, IEC 62366 specifies the application of usability engineering to medical devices [26]. Other standards referenced include defined terminology and iconography that are required on device labels (e.g., ISO 7071 [27]), standards for biocompatibility and biological evaluation of medical devices (ISO 10993 [28]), and standards for risk management which extend beyond the design of the device itself (e.g., ISO 14971 [29]). The most recent versions of these standards, as of late 2024, were used.

To ensure full compliance with IEC 60601, its requirements were implemented through a risk management process compliant with ISO 14971. This process involves defining the intended use and reasonably foreseeable misuse of the device, identifying associated hazards, and documenting appropriate mitigation strategies. For this TENS device, the identified hazards included scenarios involving incorrect stimulation levels or excessive stimulation duration. Additionally, to mitigate the risk of electric shock, all insulating materials, including the polylactic acid (PLA) housing, conductive track encapsulation, textile backing for snap connectors, and interconnecting cable insulation were selected and verified to meet the dielectric strength and flammability limits specified in IEC 60601. These risks were further addressed through the implementation of mandated hardware safety measures and user interface refinements designed to minimize the potential for user error.

To evaluate the garment feasibility and acceptability, we conducted home usability tests with individuals experiencing chronic joint pain, gathering feedback on the ease of use, comfort, electrode contact consistency, and perceived pain relief. Pain outcomes are presented as a preliminary indication of analgesic effect only, and to inform the design of a future clinical trial. Preliminary findings indicated strong user acceptance and minimal difficulties in applying garment-based stimulation, suggesting that a wearable TENS approach could improve adherence for long-term pain management in osteoarthritis patients. Lastly, we outline the design and objectives of an upcoming clinical trial, which will more rigorously assess the garment effectiveness in reducing joint pain, improving functional outcomes, and enhancing quality of life in a larger patient population.

The remainder of this paper is organized as follows: Section II presents the system architecture and design, Section III covers the software architecture, Section IV describes the home usability tests, Section V outlines the MHRA and HRA approvals process and the planned clinical trial, Section VI offers a critical discussion of implications and limitations, and Section VII provides conclusions.

## II. System Architecture and Design

**FIGURE 2.**
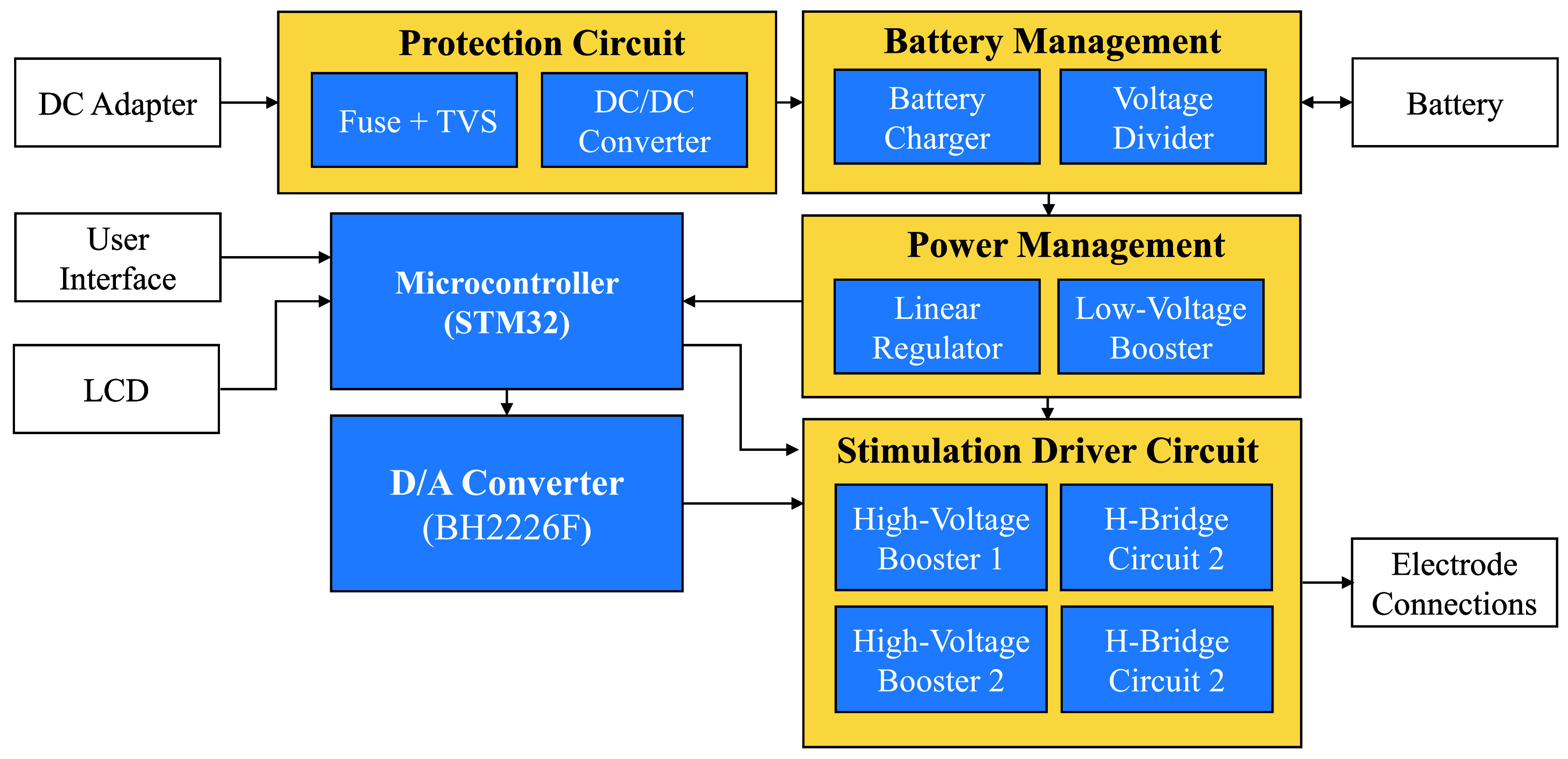
System-level block diagram of the TENS device hardware architecture, featuring a protection circuit, battery management, power management, microcontroller (*STM32*), D/A converter (*BH226F*), and stimulation driver circuits with high-voltage boosters and H-bridges. The user interface and LCD provide control inputs, while the device is powered via a DC adapter or battery.

Fig. 2 presents the block diagram of the TENS system, which consists of six key subsystems: (i) a protection circuit, (ii) a battery management circuit, (iii) a power management circuit, (iv) a microcontroller and digital-to-analog converter (DAC), (v) a stimulation driver circuit, and (vi) a user interface. A lithium-ion battery, rechargeable via a micro USB port, powers the device, while electrodes connect to the outputs of the stimulation driver circuit. Tactile switches serve as the primary means of user interaction, enabling the adjustment of device settings and parameters which are displayed on the LCD. The following subsections detail how each subsystem contributes to the safe, effective, and user-friendly management of osteoarthritis-related joint pain.

### A. Protection Circuit

The system uses a micro-USB port for charging, designated by IEC 60601 as its primary power input, which feeds into a protection circuit that incorporates (i) a 5 A fast-acting fuse to interrupt current overloads, and (ii) a transient voltage suppressor (TVS) diode with a 12 V clamping voltage to mitigate overvoltage transients. The protected power line then feeds an isolated DC/DC converter (single-output, 5 V to 5 V) [30], which provides robust 5 kVAC isolation between the USB and system grounds. This design meets the IEC 60601 requirement for 2 MOPP, maintains leakage current below $\mathrm {100~\mu \text {A} }$ during normal operation, and offers reinforced insulation consistent with medical-grade electrical isolation standards. This prevents the risk of any additional electrical circuits other than the desired path via the stimulation electrodes.

**IEC 60601 Considerations Protection Circuit:**

1) **Over-Current Protection:** The integrated fuse complies with IEC 60601 requirements by preventing electrical hazards through immediate circuit interruption during current overloads.
2) **Incorrect Power Supply:** The TVS diode safeguards against improper power source connections (e.g., out-of-specification voltage inputs) or electrostatic discharge (ESD) events, mitigating both device failure risks and potential patient safety concerns.
3) **Isolation Safety (2 MOPP):** The DC/DC converter ensures robust electrical safety through multiple protection measures: it provides reinforced insulation rated for a 5 kVAC withstand voltage for 1 min, maintains a minimum 7 mm creepage distance between primary and secondary circuits, and implements double insulation between patient connections and mains power sources.
4) **Electrical Safety:** The PCB layout implements critical safety measures through robust physical isolation. High and low voltage sections maintain a minimum clearance of 7 mm or alternatively employ 3 mm slots, effectively preventing arcing and ensuring compliance with IEC 60601-1 creepage and clearance requirements.
5) **Leakage Current Limits:** The design incorporates a leakage current control measure, maintaining ground leakage currents below $\mathrm {100~\mu \text {A} }$ during normal operation.

### B. Battery Management

The battery management system is based around a Texas Instruments *BQ25170* charging circuit, which provides a programmable charge voltage ( ${\mathrm {4.2~\text {V}}}~\pm 0.5\%$) and fast charge current (up to 800 mA). A voltage divider circuit enables real-time battery voltage monitoring through the microcontroller’s ADC input. The charger implements a three-state LED indication system: (i) continuous illumination during charging, (ii) off-state when charging is complete, and (iii) 1 Hz blinking to indicate fault conditions such as overvoltage or thermal overload. The system uses a rechargeable 3.7 V lithium-ion battery (nominal voltage) with 2 Ah capacity [31], compliant with the IEC 62133-2:2017 safety standards for secondary lithium cells.

**IEC 60601 Considerations Battery Management:**

1) **Battery Safety:** The dedicated battery charging circuit is equipped with overvoltage protection, overcurrent protection, short-circuit protection, and thermal monitoring, ensuring compliance with medical-grade safety standards.

### C. Power Management

The power management system comprises two key components: (i) a 3.3 V linear regulator [32] that provides stable power to the microcontroller, DAC, and other 3.3 V components; and (ii) a low-voltage boost converter that steps up the nominal 3.7 V battery voltage to 12 V. This intermediate 12 V rail then feeds subsequent high-voltage conversion stages, ultimately generating the therapeutic stimulation voltage of up to 130 V through additional boost conversion, as shown in Fig. 4.

**FIGURE 3.**
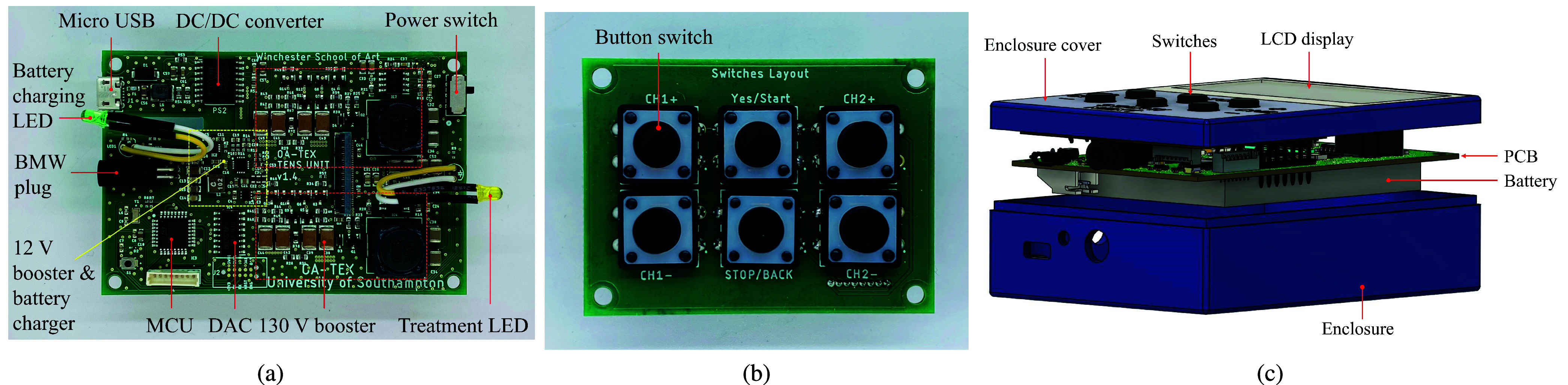
(a) Printed circuit board (PCB) of the TENS device, (b) PCB design for the button switches that control the TENS unit’s stimulation level, and (c) a 3D computer-aided design (CAD) model of the complete TENS device.

**FIGURE 4.**
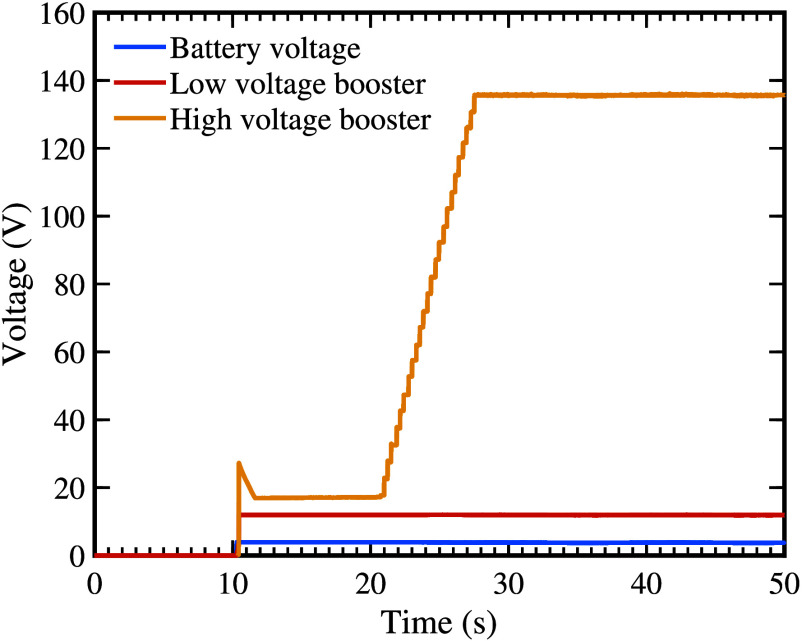
Voltage output comparison of the TENS device power supply system. The plot shows the battery voltage (3.7 V), stepped-up low voltage (12 V) from the first boost converter, and final variable high voltage output (20 to $\sim {\mathrm {130~\text {V}}}$) from the second boost converter over time. The two-stage voltage boosting architecture enables efficient power delivery for TENS while maintaining safety compliance.

**IEC 60601 Considerations Power Management:**

1) **Stable Therapeutic Stimulation Voltage:** The high-voltage booster requires a minimum operating voltage of 8 V. By providing a 12 V rail, the system can tolerate ±10% input fluctuation without compromising the required therapeutic voltage level. This design choice ensures consistent treatment delivery and adherence to Clause 201.12.4.101 of 60601-2-10 for medical electrical equipment.

### D. Microcontroller and Digital-to-Analog Conversion

The *STM32* microcontroller [33] serves as the central processing unit, executing firmware to regulate stimulation parameters such as pulse frequency and amplitude. Details of the firmware are provided in Section III. It interfaces with the digital-to-analog converter (DAC) [34] via a Serial Peripheral Interface (SPI) to generate analog control signals. These signals serve as reference inputs for the closed-loop feedback system of the high-voltage boost converter, adjusting the output voltage to produce the desired therapy patterns.

**IEC 60601 Considerations Microcontroller and Digital-to-Analog Conversion:**

1) **Output Amplitude Control:** The DAC-generated reference voltages provide continuous, linear control of stimulation amplitude from minimum to maximum output (0–130 V), satisfying Clause 201.12.1.101 of IEC 60601-2-10 for TENS devices. This ensures precise dose delivery across the entire therapeutic range while maintaining safety margins.

### E. Stimulation Driver Circuit

**FIGURE 5.**
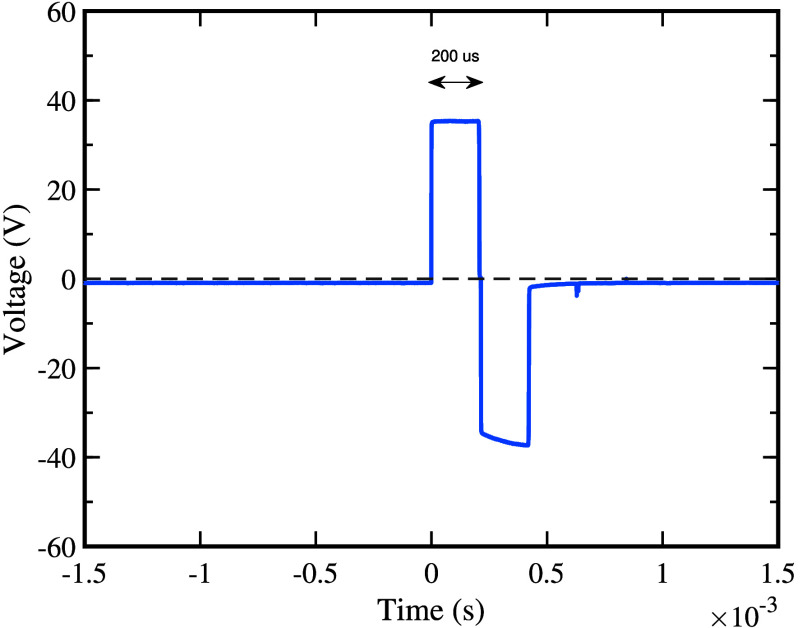
Biphasic output waveform of the stimulation driver circuit. The amplitude is adjustable via the user interface’s tactile switch.

The stimulation driver circuit integrates two critical subsystems to deliver controlled therapeutic pulses: (i) a high-voltage boost converter and (ii) an H-bridge output stage. The boost converter efficiently steps up the 12 V to the required therapeutic range (14–130 VDC) [35]. The boost converter is then coupled with an H-bridge circuit to generate biphasic pulses for muscle stimulation (Fig. 5). The circuit utilizes a combination of NPN [36] and PNP [37] transistors, controlled by the microcontroller. The design incorporates $\mathrm {300~\Omega }$ current-limiting resistors at the output terminals, providing robust short-circuit protection, ensuring patient safety and IEC 60601 compliance.

**IEC 60601 Considerations Stimulation Driver Circuit:**

1) **Output Voltage Control:** The high-voltage boost converter employs feedback circuitry to maintain a stable, user-prescribed output voltage. This feedback loop ensures that the output voltage never exceeds safe limits set by IEC 60601, preventing excessive electrical energy being delivered to the patient.
2) **Short-Circuit Protection:** Resistors in the H-bridge output path provide a current-limiting mechanism. In the event of a short circuit or fault condition, these resistors restrict the current to a safe level, protecting both the circuit and the patient. The resistors also protect the boost converter and H-bridge transistors from excessive current flow, mitigating potential circuit damage and ensuring reliable operation over the device’s lifetime.

### F. User Interface

To enable intuitive interaction, the system incorporates tactile switches and a liquid crystal display (LCD) [38]. Patients and clinicians can use the tactile switches to adjust intensity or the treatment duration of two preconfigured stimulation modes, and initiate or terminate therapy sessions. The LCD provides real-time feedback on parameters such as stimulation intensity, battery status, and session duration, thereby enhancing usability and promoting adherence in both clinical and home environments.

**IEC 60601 Considerations User Interface:**

1) **Operator controls and indication:** The interface features clear labeling, such as Start/Stop and Increase/Decrease intensity buttons, along with LCD feedback, ensuring users can easily understand the operational status of the device.
2) **Treatment start indication:** A yellow LED indicates that the treatment has started and is in progress, satisfying Clause 201.12.4.103 of IEC 60601-2-10 for TENS devices.
3) **Battery charging indication:** A green LED is used to show that the battery is charging.
4) **Lock-out/override features:** Software logic is implemented to temporarily lock critical parameters during therapy, preventing unintentional changes through inadvertent button presses, once stimulation is active.

### G. Device Enclosure and Mechanical Safety

The TENS unit’s electronic components are housed within a durable enclosure designed to protect internal circuitry from external impact, moisture, and environmental stress. The enclosure is 3D printed using PLA plastic [39]. The enclosure is contoured to eliminate sharp edges and facilitate comfortable handling by users.

**IEC 60601 Considerations Device Enclosure and Mechanical Safety:**

1) **Dielectric Strength (Subclause 8.8.3):** Under IEC 60601-1:2006 + A2:2021, solid insulation in medical devices must withstand dielectric-strength test voltages of 2 kV for operator protection and 3 kV for patient protection to satisfy the 2 MOPP requirement. Because the TENS unit’s peak operating voltage lies between 71 V and 212 V, all insulating materials including the PLA housing, garment conductive track encapsulation, textile backing for snap connectors, and interconnecting cable insulation were verified against these limits. Before testing, all samples were pre-conditioned in a humidity chamber according to section 5.7 of the standard ( $93~\pm 3$% RH and room temperature) for 168 hours. Dielectric testing was performed with a Variac-controlled high voltage transformer (25 kV, 50 VA) that delivers a continuous 50 Hz AC voltage; the output was monitored using a multimeter connected via a 1:1000 high-voltage probe. The system features a $100~\pm {\mathrm {10~\text {m}\text {A} }}$ electronic current trip. This corresponds to approximately 1 mA on the high-voltage side, which simultaneously serves as an indicator for electrical breakdown conditions. A test cell with a lower 25 mm diameter and an upper 20 mm diameter polished brass electrode, conforming to IEC BS EN60243-1 standards, was used to ensure consistent electrode geometry and minimize edge effects.
2) **Push Test (Clause: 15.3.2):** In compliance with IEC 60601-1:2006+A2:2021 Clause 15.3.2, the enclosure must demonstrate sufficient rigidity to protect against unacceptable risks. The test evaluates the mechanical strength of the enclosure under applied force to ensure it maintains basic safety and essential performance. Experimentally, the device was subjected to a steady force of ${\mathrm {250~\text {N}}}~\pm {\mathrm {10~\text {N}}}$ for 5 s using a calibrated machine. A 25 kg weight was used to achieve the force, applied over a 30 mm diameter circular planar surface to ensure consistent testing conditions.
3) **Impact Test (Clause 15.3.3):** In compliance with IEC 60601-1:2006+A2:2021, the enclosure must demonstrate sufficient resistance to impact to protect against unacceptable risks. This test evaluates the mechanical strength of the enclosure to ensure it can withstand mechanical stress without compromising basic safety or essential performance. Experimentally a solid, smooth steel ball with a diameter of approximately 50 mm and a mass of ${\mathrm {500~\text {g}}}~\pm {\mathrm {25~\text {g}}}$ is used for the test. The ball is allowed to fall freely from a height of 1.3 m onto the surface of the device to simulate vertical impacts. Additionally, the steel ball is suspended by a cord and swung as a pendulum to apply a horizontal impact against each relevant part of the device.
4) **Drop Test (Clause: 15.3.4):** In compliance with IEC 60601-1:2006+A2:2021, the enclosure must withstand mechanical stress caused by free falls to ensure it does not result in an unacceptable risk. The test simulates typical impacts encountered in clinical and home environments, such as accidental drops from a handheld height or a treatment table. Experimentally, the device is subjected to a free fall from a height of 1 m onto a ${\mathrm {50~\text {m}\text {m} }}~\pm {\mathrm {5~\text {m}\text {m} }}$ thick hardwood board which is placed flat on a concrete surface. The test is conducted in multiple orientations (e.g., face-down, edge, and corner) to evaluate the enclosure’s structural integrity under varying impact conditions.
5) **Ingress Protection (IP22 Conformance):** In compliance with IEC 60529, the enclosure is designed to meet a minimum IP rating of IP22 or higher to protect against intrusion by solid objects of 12 mm or more and dripping water when tilted up to 15°. This is critical for preventing accidental fluid ingress, which could lead to short circuits or shock hazards, particularly in environments where minor physical interference or occasional water exposure may occur. To verify IP22 conformance, the enclosure was subjected to two tests:
  • **Solid Object Test:** A calibrated probe with a diameter of 12 mm was used to determine if any part of the object could penetrate the enclosure. The sample was positioned as it would be during normal use.
  • **Dripping Water Test:** The test sample was placed on a tiltable platform inclined up to 15 and exposed to vertically dripping water from a height of 200 mm for 10 min. This simulated real-world conditions, as outlined in Fig. 3 of IEC 60529, to ensure consistency and accuracy.

### H. Wearable Electrodes and Integration

The garment integrates four textile electrodes ( ${\mathrm {50~\text {m}\text {m} }}\times {\mathrm {50~\text {m}\text {m} }}$) positioned to target the knee joint for osteoarthritis pain management. The electrode pads and conductive tracks were designed as layered, vector files in Adobe Illustrator and printed by Conductive Transfers Ltd. (Barnsley, UK). These were applied to the garment’s base fabric using a pneumatic heat press, and silicone–carbon dry electrodes (Fabink E-0002) were stencil-printed on top. This formulation was selected for its soft, flexible properties and slightly tacky surface finish, which improves skin adhesion without requiring additional adhesives. Conductive snaps at the end of each track allow secure connection to the stimulation unit via an interconnecting cable.

The garment body is fabricated from a 4-way stretch knit fabric supplied by Whaley’s Bradford Ltd. (Bradford, UK), with a two-way zip running from calf to hip to facilitate easy donning and doffing for users with limited mobility. The design ensures consistent electrode alignment over the knee, addressing a key limitation of conventional TENS systems that rely on manual electrode placement.

Prior to each treatment session, users are required to lightly moisten the electrode areas and surrounding skin using a spray bottle filled with water to reduce impedance and enhance conduction for effective current transfer. This hydration step is essential for comfortable stimulation and eliminates the need for conductive gels or saline sprays, supporting ease of use and garment reusability.

The novelty of this garment design lies in its combination of ease of use, washability, and reusability, alongside consistent electrode placement that simplifies setup while maintaining stimulation quality. These features make the system practical for long-term home use and reduce environmental impact compared to disposable gel electrodes. Wash testing of the garment-integrated electrodes was conducted on a commercial Electrolux W475H machine. Each cycle ran at $30~^{\circ }$ C for 27 min with a 1000 rpm spin; one non-biological Persil liquid detergent capsule was used per cycle. After each cycle, samples were hang-dried overnight at room temperature, and electrical resistance was measured only after complete drying. Testing was performed over five consecutive cycles for two electrode pairs (Channel 1: E1–E2; Channel 2: E3–E4). As shown in Fig. 7, resistance increased with cycle count, yet remained below the pre-specified design limit for safe, effective stimulation.

**FIGURE 6.**
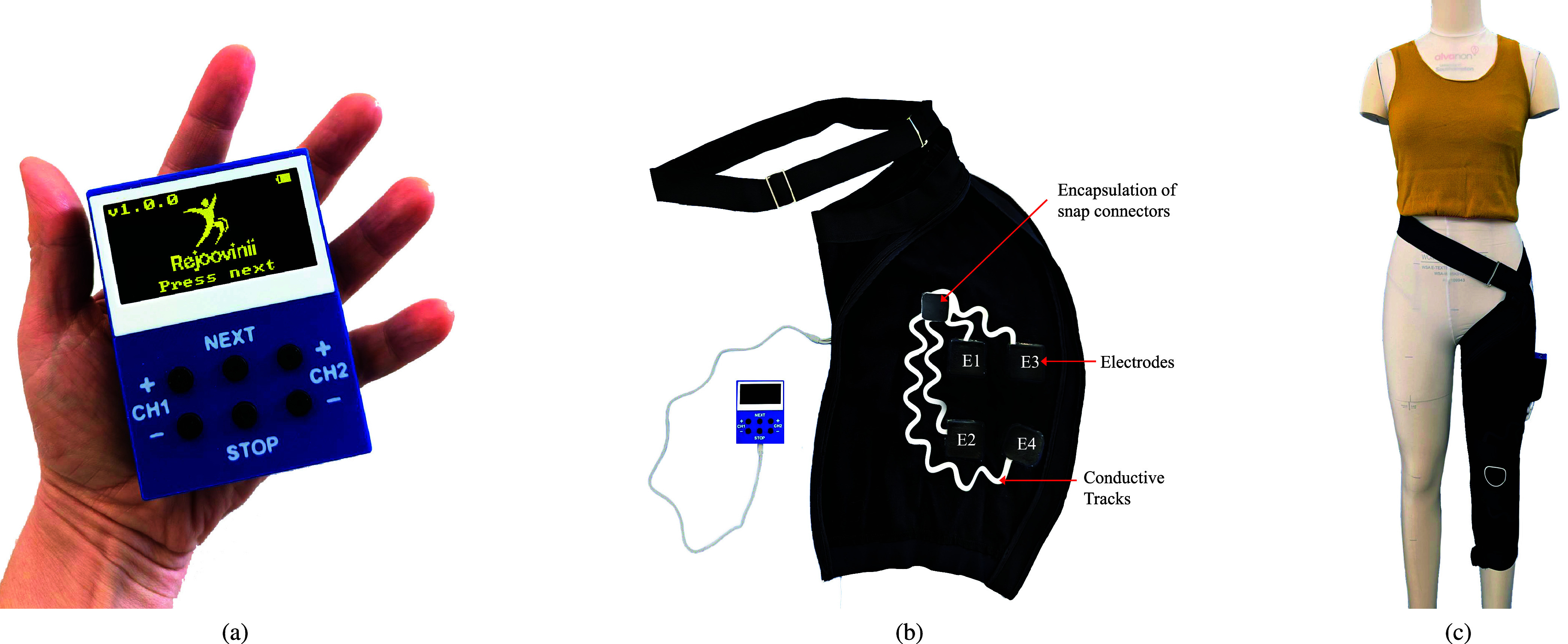
(a) TENS electronics in a handheld enclosure; (b) integrated textile garment with four printed electrodes, Channel 1 (E1-E2) and Channel 2 (E3-E4), connected to the TENS unit via laminated conductive tracks; the fifth upper square is an encapsulation material for snap connectors; (c) final wearable design demonstrated on a mannequin.

**FIGURE 7.**
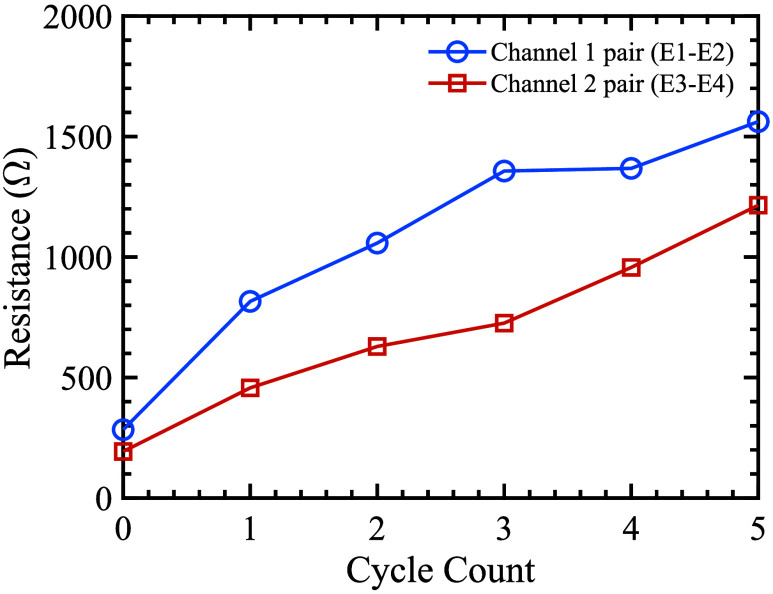
Electrode resistance versus wash cycles for Channel 1 pair (E1–E2) and Channel 2 pair (E3–E4). Resistance increased with laundering but remained sufficiently low for effective stimulation.

### I. Translational Process in an Academic Setting: Challenges and Pragmatic Solutions

Designing a medical-grade wearable device within an academic environment, without the quality-management infrastructure typically available in industry, presents significant translational challenges. Reflecting on our development pathway, we identify several key obstacles and the pragmatic strategies employed to overcome them as a small academic team. These insights are intended to serve as a practical blueprint for other researchers undertaking similar translational projects. Moving from early prototypes to a near-final device required multiple design iterations while managing the risk of component shortages and discontinuations. To address this, we adopted conservative bill-of-materials planning, selecting components with good long-term availability and pre-ordering sufficient quantities once the architecture stabilized. Without in-house manufacturing capabilities or an established supply chain, we relied on external partners for PCB fabrication and component assembly, aligning our design with their available component inventory to control cost and lead time for small-batch builds, while ensuring the PCB supplier was certified for medical device fabrication.

Designing a functional wearable system introduced distinct challenges. To ensure reliable electrode-skin contact without compromising the flat, seam-free surface required for laminated electrodes and interconnects, we developed a one-piece outer panel using four-way stretch fabric with negative ease and contour pattern lines. This design uses gentle compression and strategic stretch to conform to the knee anatomy. To accommodate diverse body types, the garment was co-designed and graded into seven sizes with adjusted electrode positions, validated through fittings with a representative user group. An ergonomic double-ended zip with anti-pinch guards and large pulls was incorporated to address donning and doffing difficulties for older users or those with limited mobility, with the design refined iteratively through end-user testing.

Ensuring IEC 60601 compliance within a small academic team was particularly demanding. Our strategy was multi-faceted and proactive. From the outset, key safety considerations including creepage and clearance distances, insulation strategy, leakage current limits and 2 MOPP architecture were embedded into the circuit and mechanical design, rather than retrofitting them at the end of development. To access specialized testing, we collaborated with other university research groups for mechanical and dielectric assessments and engaged a medical device consultant to review our design and compliance strategy, ensuring our development remained aligned with regulatory requirements.

Finally, a core translational objective was to enable independent home use with minimal professional oversight. In contrast to complex, configurable commercial units, we intentionally simplified the user interface based on feedback from early prototypes. The final device allows control only over stimulation intensity and treatment duration, with all other therapeutic parameters fixed. This design-for-simplicity paradigm, validated in our usability study by high scores for ease of setup and independent operation, represents a critical translational decision to bridge the gap between clinical efficacy and practical, sustained patient adherence in a home-care environment.

## III. Software Architecture

The device’s operation is orchestrated by an *STM32G031K8* microcontroller. This is a general-purpose ARM Cortex microcontroller with 64 kBytes of program memory and 8 kBytes of RAM. The microcontroller interfaces with the majority of the other components in the system, processing user input and configuring the hardware into the desired states and managing the timing of the resultant stimulation pulses.

The development process for the chip’s firmware followed the requirements of IEC 62304, a standard written to regulate the design, development and deployment of software for medical devices and which is required by IEC 60601. Within this standard there are levels of requirements based on the safety classification. For this device, the firmware was determined to be class B, indicating the software can contribute to a hazardous situation resulting in non-serious injury. Initially, precise software requirements were gathered and documented. This included specifications for the stimulation output as well as the requirements of the user interface and the other electronic components with which the microcontroller interfaces. These requirements were then used to guide the development of the firmware.

The firmware was written in $C$, which is the most widely supported programming language for microcontrollers. Guidelines for the use of $C$ in safety critical systems have been developed to reduce the potential for unexpected behavior. In this case, the MISRA $C$ guidelines [40] were followed, and enforced by automated testing. The microcontroller provided some libraries as an application programming interface (API) to the chip’s hardware functionality. This was tested through the same processes to ensure that it functioned as expected. To ensure the software operates as expected, tests of its functionality, both of individual parts of the code and of the system as a whole (unit and integration tests) were also written, ensuring that each component performed as expected and the software fulfilled all the specified requirements. Each test was linked to one or more requirements, so it was possible to confirm that all requirements were validated. For each software release all the tests should be carried out and the results documented.

**FIGURE 8.**
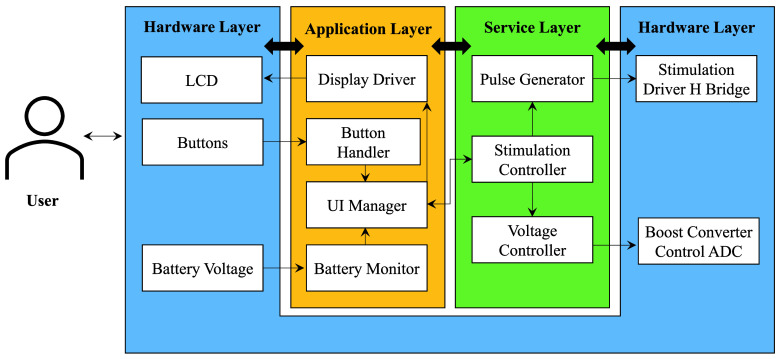
Structure of the microcontroller firmware showing each of the modules in use and the flow of data between the components.

Fig. 8 shows the overview of the modules each of which manage one part of the system. Implementation as separate modules allows for easier testing and validation of each component as it can be directly controlled in the unit tests. The hardware reset state of the chip has all the outputs in a safe idle state in which the stimulation outputs are disabled and power supplies deactivated. The software initially will configure the system before entering a loop which goes through each block and processes any required actions from each module in turn. Items with tight timing requirements such as registering button presses or starting an output sequence are triggered asynchronously via interrupts. A watchdog component runs in the background to reset the chip should the program stop for any reason, mitigating the safety risks from this situation.

The overall flow of the system is controlled by the user interface module, this module responds to the user inputs and navigates through a set of states for the user to setup the stimulation profile. The display contents are generated from this state and information from other modules, such as the battery monitor and stimulation controller, as required, before being passed onto the display driver which updates the screen with the new data. The settings selected by the user are also passed to the stimulation controller module which handles the high-level control of the stimulation profile such as strength and treatment duration. Delivery of the pulses is handled by the pulse generator; the start of a pulse sequence is triggered via interrupt rather than using the processing loop to keep consistent timings. The timings of the pulses need to be even more tightly controlled so the pulse generator will block other interrupts and not perform any other processing during this window. As the pulses are short this has a negligible impact on the responsiveness of the rest of the system.

**IEC 60601 Considerations Software:**

1) **IEC 62304 defined development process:** Prescribes well-defined software requirements each with an associated test plus a defined procedure for software deployment. This ensures that all the features that are necessary for the stimulator to function safely are present.
2) **Coding guidelines and testing:** Thorough testing of the software at multiple levels against the requirements and using safely written code in compliance with the MISRA C guidelines reduces the likelihood of bugs affecting the software.
3) **Fail-safes:** Multiple mechanisms are used to prevent patients from receiving any form of unexpected stimulation. These include the above-mentioned handling of pulse timing which prevents other events from affecting the duration of stimulation pulses. A watchdog timer is also used which resets the device if an error has caused it to stop responding for more than 20 ms, and the system is designed such that when it resets, because of the watchdog timer, a power failure or otherwise, it returns to its safe idle state and will not output any stimulation.
4) **Audited SOUP:** IEC 62304 requires that software of unknown provenance (SOUP) is held to the same standards as the rest of the software system. In this case the only external software is the hardware abstraction layer provided by STMicroelectronics for use with the microcontroller. This is a relatively small amount of code which is available in source form, allowing it to be inspected and it is included in the testing procedure to ensure that it functions as expected.

## IV. Home Usability Tests

### A. Participant Demographics

Eleven adults with osteoarthritis knee joint pain (designated as participants P–01 through P–11) were enrolled in the study. Ethics committee approval from the University of Southampton [ERGO II number: 77286] was obtained for the co-design activities and home usability testing conducted in this study. The home usability testing is part of the design and development conducted to gain additional feedback from the users to refine the device before a future clinical investigation which requires MHRA and HRA approvals. Participants were recruited from a community setting, with inclusion criteria requiring: (i) aged between 45 and 70; (ii) diagnosed with knee osteoarthritis by a healthcare professional or able to complete a self-assessment survey to evaluate eligibility; (iii) knee pain which impacts on daily activities; (iv) able to attend two sessions in person and (v) willingness and capacity to provide written consent. Exclusion criteria included the following: (i) Having an active device implant (e.g., pacemaker); (ii) having a partial or total knee replacement; (iii) having a stent in the lower limb; (iv) having uncontrolled epilepsy; (iv) being pregnant or planning to become pregnant, or breastfeeding and (v) having skin sensitivities or sensation problems. The authors confirm that informed consent was obtained. The recruited end user group including two men and eight women, aged 50–70, represented a range of body types from *XS* to *2XL*.

### B. Study Protocol

Participants were instructed to use the TENS garment according to a standardized seven-day protocol specifying a daily treatment duration of one $\mathrm {30~\min }$ session with the option of an additional $\mathrm {30~\min }$ session on the same day. Pain scores were assessed at baseline and post-intervention, permitting calculation of both absolute and relative reductions as summarized in Table 1. The percentage reduction in pain was calculated as:

\begin{equation*} \mathrm {Reduction \; (\%)} = \frac {\Delta }{\text {Pre-treatment pain}} \times 100 \tag {1}\end{equation*}

where $\Delta $ represents the difference between the pain score recorded before treatment and the pain score recorded after treatment. Participants recorded their experiences in a daily test diary, noting knee pain levels before and after TENS treatment on a visual analogue pain score (VAS), any irritation or redness at the site of electrode contact, session duration, stimulation levels and any other relevant information such as activities performed during TENS treatment/use, and any changes in their stiffness or flexibility.

**TABLE 1:** Summary of Pain Reduction Outcomes from the Seven-Day Home Usability Study. The Last Row Shows Group Averages. The Reduction (%) Value of 54.79% is the Mean of the 11 Individual Percentage Reductions. If the Percentage Reduction is Instead Calculated from the Group-Mean Pain Scores (3.45 Pre-Treatment and 1.86 Post-Treatment), the Overall Reduction is 46.1%

User ID	Pre-treatment pain	Post-treatment pain	Absolute reduction	Reduction (%)
P–01	4.6	2.8	1.8	39.1
P–02	3.3	2.0	1.3	39.4
P–03	3.3	1.1	2.2	66.7
P–04	2.3	1.2	1.1	47.8
P–05	2.8	2.3	0.5	17.9
P–06	1.8	0.5	1.3	72.2
P–07	1.4	0.1	1.3	92.9
P–08	2.4	1.3	1.1	45.8
P–09	6.9	3.0	3.9	56.5
P–10	8.2	6.2	2.0	24.4
P–11	1.0	0.0	1.0	100.0
Average	3.45	1.86	1.59	54.79

### C. Device Setup and Adherence

Prior to the study, participants attended at least one in-person fitting session to ensure they received a garment of the correct size, with appropriate electrode contact and with electrodes aligned precisely over the knee joint. Participants were trained on how to use the TENS garment, operate the user interface, and adjust parameters within a prescribed range, as well as correct application of the electrode moistening protocol. A hard copy of the instructions for use, covering both the garment and TENS unit, was given to participants for reference during the home usability test. Adherence was monitored via self-reported usage logs, which indicated that all participants followed the protocol for the seven-day study period.

### D. Pain Relief Results

The home usability test and associated pain relief data were collected as part of an early-stage usability and technical feasibility evaluation rather than a clinical investigation. The primary objective was to assess garment fit, usability, comfort, and integration into daily life, not to establish definitive clinical efficacy. Pain outcomes reported here are exploratory and presented as preliminary indications of potential analgesic effect, intended to inform the design and methodology of any future clinical investigations.

The intervention demonstrated an average absolute pain reduction of 1.59 points, corresponding to a 54.79% improvement from baseline across the seven-day evaluation period. Individual responses varied considerably, ranging from minimal improvement (17.9%) to substantial relief (100%). While these preliminary findings suggest a potential analgesic benefit, they should be interpreted with caution. Osteoarthritis symptoms can fluctuate due to factors such as activity level, time of day, and seasonal variation, and the small sample size (n = 11) limits generalizability. In this study, each participant acted as their own control, which helps mitigate the influence of natural variability. These results therefore represent early indications rather than definitive evidence of efficacy. To rigorously establish clinical benefit, a randomized controlled trial with an appropriate comparator (e.g., sham intervention or blinded on/off design) and sample size justification is planned. This future trial will provide a more robust assessment of pain outcomes, functional improvements, and adherence under controlled conditions for long term use (e.g., $\geq 12$ weeks).

### E. Observations on Safety

No serious adverse events or device malfunctions were reported during the study period, although issues related to minor skin irritation, garment durability and design were noticed. Wet gel electrodes typically experience levels of skin irritation of about 3% [41]. In the case of the presented wearable the electrodes are dry but the user is asked to spray water on the skin and electrodes before donning the garment and performing the treatment. However, in the home usability trial, three out of eleven users experienced some redness and irritation at the site of electrode contact, but without any broken skin or itching. It should be noted that other studies have demonstrated that one third of TENS users may develop skin irritation at the site of electrode contact, which can be managed with standardized skin care and topical corticosteroid steroid treatment [42]. A future trial will further evaluate skin irritation, but it is expected that an electrode gel (e.g., Spectra 360) will be applied to the electrodes instead of spraying water with the target of reducing skin irritation by creating a thin barrier between the electrode and the skin.

These findings demonstrate that the garment-based system was well tolerated among study participants. Issues with garment durability were reported, particularly related to damage to the printed conductive tracks connecting the garment and the TENS unit. This affected functionality resulting in a reduction in stimulation strength but did not compromise user safety. This can be resolved by widening the conductive track or modifying the design to eliminate printed tracks running between electrodes, or reinforcing the tracks in this region.

### F. User Feedback and Satisfaction

Following the home usability test period, participants were interviewed one-to-one to discuss their experiences and provide detailed feedback on the comfort and usability of the garment, the instructions for use, the TENS electronic unit, cables and any issues encountered. Participant feedback highlighted satisfaction with the choice of fabric used in the garment, and the wearable design’s ease of application.

Eight out of eleven users reported that all electrodes maintained good contact with their skin. However, three users experienced occasional loss of stimulation from the electrodes on the inside of the knee. Loss of electrode contact will be addressed through the addition of wide elastic straps attached to the garment exterior to provide pressure on the electrodes.

Feedback on the garment’s fabric emphasized its comfort and breathability, although some users expressed concerns about wearing it in hot weather. Users commented positively on the fabric, describing it as soft, non-itchy, and secure. They noted that it did not cause sweating, felt breathable, and its elastomeric properties allowed it to move naturally with the body. Users acknowledged that the fabric is thick and strong but understood the necessity. Two users mentioned that the fabric felt slightly warm during hot weather but found it acceptable since it only needed to be worn for $\mathrm {30~\text {m}}~\text {to}~\mathrm {60~\text {m}}$ at a time. Users reported engaging in a variety of activities, both sedentary and non-sedentary, indoors and outdoors, while using the device during the home usability test. All users reported being able to successfully set up the TENS treatment duration and strength without difficulty, following the instructions provided. Charging the device was necessary for most users at least once during the week. No users reported any major issues with charging the device.

In terms of usability, all users were able to set up the garment and TENS unit treatment in under five minutes with 90% under three minutes, indicating good usability of the device. Initial setup time (before the home usability test) was 00:07:01, and setup time after the home usability test was 00:02:12, resulting in a decrease in setup time of 00:04:49 (68.6%) indicating good learnability of the device. All users reported that the garment fitting and TENS unit setup instructions were clear and straightforward/easy to understand. Seven out of eleven users (63.6%) indicated that they consulted the instructions only on the first or second day of use. After this initial period, they were able to put on the garment and set up the TENS unit without referring to the instructions. While not strictly a clinical experience, the combined results of lab-based testing and the home usability test provide confidence in the safety, usability, and initial efficacy of the device for further clinical testing in the future pilot randomized control trial.

## V. MHRA and HRA Approvals for Clinical Investigation

To progress towards clinical validation of the e-textile TENS device, approval from the UK MHRA and HRA were sought to conduct a pre-market pilot randomized control trial (RCT). As this device qualifies as an investigational medical device and is not yet CE/UKCA-marked, under UK regulations, MHRA [43] approval was required before a clinical trial, using human participants, could proceed. This was to ensure participant safety, confirm that the device met essential safety and performance criteria, assessment of the clinical investigation plan and authorized the trial facilities, staff and sponsor.

The MHRA application required submission of a clinical investigation plan, clinical investigation brochure, device technical documentation, history of clinical evaluation to date, risk management files, instructions for use, and evidence of conformity with applicable electronic, medical device and usability standards. To support the application, it was demonstrated that the system met essential performance and safety criteria through a combination of lab-based bench testing and in-home usability testing. Risk mitigation strategies and human factors considerations were also documented, specifying how they were incorporated into the overall design of the device. Labeling and instructions for use reduced the likelihood of misuse and ensured user safety during the trial.

The HRA assessment focuses on ethics and governance to protect the rights, safety, dignity and wellbeing of research participants while promoting ethical research. The HRA application is reviewed by a local Research Ethics Committee (REC). The REC provided detailed reviews of the Clinical Investigation Plan, Participant Information Sheet, Consent Form, Recruitment Materials, Follow-up Questionnaires, Device Labels, Instructions for Use, Investigator CVs, Sponsorship and Insurance documentation.

The future pilot RCT will evaluate the clinical investigation protocol and device’s effectiveness in reducing joint pain, improving functional mobility, as well as obtaining qualitative data on device usability, any improvements in quality of life, device durability, skin irritation and feedback to improve the device. The results of this pilot RCT are a key step towards gathering clinical evidence to support a future definitive RCT and commercialization of the device.

## VI. Discussion

A custom-designed TENS device was essential for this study to meet technical and safety requirements not universally available in commercial units, including high-output, dual-mode stimulation, and compliance with enhanced 2 MOPP safety standards for at-home clinical use. Furthermore, the device was deliberately simplified to minimize user errors among older adults and optimized in form factor for integration into a wearable garment, with tailored placement of charging and electrode ports and switches. Crucially, the device incorporates a sham mode to enable blinded comparisons in a randomized controlled trial.

This research addresses a distinctive translational question: *Can a clinically effective TENS device for osteoarthritis be designed to be simple, wearable, and suitable for a home-care environment with minimal professional oversight?* This necessitated a custom system that combines, in a single regulatory-ready design, features not jointly available in existing commercial or research devices, including a deliberately simplified, user-centric interface (stimulation level and time only) integrated with high-output stimulation capability; a regulatory-compliant device developed with a translational blueprint in accordance with IEC 60601-1 and ISO 14971; and a co-designed, robust wearable system intended to support long-term adherence. The early-stage home usability study provides initial validation of these requirements, demonstrating high ease of use and setup, independent operation without professional support, and preliminary evidence of analgesic benefit, thereby positioning the device as a translational step that addresses a gap in both the literature and the current product landscape.

The intention is to progress this device until it is suitable for clinical use. Although this device initially targets the UK market it complies with the relevant international standards. This paper contributes to knowledge on how to design and develop medical device to transfer healthcare from the hospital to the home using wearable technologies. So clinical use will be primarily at home outside the clinic. The device therefore needs to satisfy the relevant standards relating to both home use and the Medical Device Regulations, in order to obtain MHRA and HRA approvals for a future clinical trial. Early-stage design choices are required to satisfy these standards and the contribution to knowledge that this paper describes are the key requirements impacting the design from the standards and the solutions we adopted thus informing other academic researchers and product developers. In addition to new knowledge on design and development for regulatory compliance, this paper also introduces technical advances in e-textile wearable technologies with dry electrodes for wearable electrotherapy applications that are comfortable to wear, easy to use, and washable/reusable.

The work presented here should be viewed as a translational step, an early-stage evaluation of a safety-compliant wearable device in real-world home use, designed to bridge the gap between lab-based prototypes and a clinical trial.

## VII. Conclusion

This paper presents the design and preliminary evaluation of a wearable TENS garment compliant with relevant regulatory standards (e.g., IEC 60601) for managing osteoarthritis-related joint pain. The system incorporated multiple technical innovations, including robust power management, high-voltage drive circuits, and an intuitive user interface, to meet both safety and usability requirements. In addition, the garment integrates silicone-carbon dry electrodes offering a washable and reusable design that ensures consistent electrode alignment and ease of donning and doffing.

Initial home usability testing with eleven participants demonstrated promising indications of pain relief and improved knee flexibility, with most users reporting notable reductions in pain and no serious adverse events. These findings should be interpreted as preliminary and indicative only, as the study was an early-stage usability evaluation for device refinement rather than a formal clinical investigation. The small sample size and variability inherent in osteoarthritis symptoms further limit generalizability. The novelty of this work lies in its patient-centric design and its potential to overcome key limitations of conventional TENS systems, such as the challenges of accurate manual electrode placement and reliance on disposable gel pads.

Future research will refine the device to address issues identified in the home usability study before conducting a pilot RCT with a sham comparator to rigorously assess the protocol of the clinical investigation (e.g., randomization, recruitment, and retention, patient adherence to user guidelines) and efficacy in pain relief and other outcome measures (e.g., quality of life, device safety). The pilot RCT will inform a definitive RCT to determine clinical efficacy and cost effectiveness. Ultimately, this work highlights the transformative potential of wearable medical devices in chronic pain management, supporting scalable, home-based care and improving quality of life.
